# Analysis of the Consumption of Drugs Prescribed for the Treatment of Asthma in Belgian Children

**DOI:** 10.3390/ijerph19010548

**Published:** 2022-01-04

**Authors:** Natacha Biset, Wies Kestens, Dominique Detemmerman, Murielle Lona, Güngör Karakaya, Ann Ceuppens, Stéphanie Pochet, Carine De Vriese

**Affiliations:** 1 Department of Pharmacotherapy and Pharmaceutics, Faculté de Pharmacie, Université Libre de Bruxelles (ULB), 1050 Brussels, Belgium; natacha.biset@ulb.be (N.B.); Stephanie.Pochet@ulb.be (S.P.); 2 Department of Studies & Innovation, Mutualités Libres—Onafhankelijke Ziekenfondsen, 1070 Brussels, Belgium; Wies.Kestens@mloz.be (W.K.); Dominique.DeTemmerman@mloz.be (D.D.); Murielle.Lona@mloz.be (M.L.); Guengoer.Karakaya@mloz.be (G.K.); Ann.Ceuppens@mloz.be (A.C.)

**Keywords:** children, respiratory disease, asthma, treatment

## Abstract

(1) Asthma is one of the most common chronic diseases in the world among children. The main purpose of this study was to analyze the consumption of asthma medications in order to investigate asthma in children (2–18 years) and the association with health care consumption; (2) a retrospective study using anonymized administrative data for 2013–2018 from the third largest Belgian health insurer was conducted; (3) in 2018, 12.9% of children received at least one asthma medication and 4.4% received at least two packages with a minimum of 30 days between purchases. Preschool children (2–6 years) were three times more likely to take asthma medication than older children (7–18 years). ICS, in combination or not with LABA, were the most dispensed drugs among children. Children with asthma medications were almost twice as likely to receive antibiotics, more likely to end up in the emergency room, and twice as likely to be hospitalized; (4) most children took ICS, according to the GINA guidelines. High rates of nebulization in young children were observed, despite the recommendation to use an inhaler with a spacing chamber as much as possible. Finally, children who took asthma medications were more likely to end up in the ER or be hospitalized.

## 1. Introduction

Asthma is a common non-communicable disease of the respiratory system affecting the lower airways. It is characterized by non-specific respiratory symptoms such as wheezing, breathlessness, chest tightness or cough [1]. Asthma has physical, emotional and social impacts on the life of a child [2]. Children with asthma are more likely to be in poor health; the disease is also associated with decreased daily exercise, avoidance of social activities and increased school absenteeism [3]. Allergic mechanisms can be involved in almost half of people with asthma [4]. Furthermore, children with asthma, and especially severe asthma are more likely to receive other medications, leading to substantial higher healthcare resource use and costs than average patients without asthma [5].

The diagnosis and management of asthma in preschool children is difficult due to the number of non-specific symptoms, the difficulties in evaluating the history of symptoms, and in the usage of tests. Indeed, tests are not recommended for children under 5 years old, particularly since they are not always able to perform them [6]. Moreover, according to Brand et al., “there is little research evidence to guide the choice of investigations”. Furthermore, many wheezy phenotypes have been identified [7].

Despite many advances in asthma treatment, including monoclonal antibodies targeting immunoglobulin E or interleukins pathways, many asthma pediatric patients remain uncontrolled, which leads to significant medical costs [8]. Uncontrolled pediatric asthma is associated with high exacerbation rates, impaired quality of life, and persistent bronchial obstruction [9]. The prevalence of asthma varies from one country to another and from one part of a country to another [10]. According to Achakulwisut et al., between 7.5 and 10.0% of Belgian children present asthma, and respiratory medications account for almost half of the total number of daily doses prescribed by pediatricians in ambulatory care [11,12]. However, limited data are available on the drug consumption among children with asthma in Belgium.

The main purpose of this study was to evaluate and analyze the consumption of asthma medication in Belgian children (2–18 years). In a second step, we assessed the care consumption of children taking asthma medications in terms of allergy medication or antibiotics, emergency departments visits, and hospitalizations.

## 2. Materials and Methods

A retrospective study using anonymized administrative data for 2013–2018 from the Independent Health Insurance Funds (Mutualités Libres–Onafhankelijke Ziekenfondsen) was conducted. This database contains the anonymized socio-demographic data of its members, as well as all the medical services and pharmaceutical dispenses that have been reimbursed by the obligatory health insurance. The Independent Health Insurance Funds represents about 20% of the whole Belgian population, and it is the third largest insurer in Belgium.

For the first purpose, we selected from our database Belgian children aged between 2 and 18 years old. Children younger than 2 years old were excluded due to the high prevalence of bronchiolitis treated with anti-asthmatic medications. In order to identify children with asthma, we used asthma medication dispensing, as we do not have diagnostic data or functional test results. The asthma medications selected were SABA (Short-Acting Beta Agonists), SAMA (Short-Acting Muscarinic Antagonist), ICS (Inhaled Corticosteroids), LABA (Long-Acting Beta Agonists), montelukast, sodium cromoglicate, theophylline, omalizumab, and drug combinations of ICS-LABA and SABA-SAMA. All these asthma medications required a prescription in Belgium. Two approaches based on the drugs dispensing were used: (1) The first approach consisted of selecting children with at least one dispense of an anti-asthmatic drug. It allows broad identification of all children with respiratory problems such as asthma. (2) The second approach included children with at least two deliverances of anti-asthmatic drugs with at least 30 days between two deliveries. This approach allows us to identify children with a more severe clinical picture. The children included in this second approach were also included in the first one. These two groups were compared to a third group that included children who had not received asthma medication.

For the second purpose, the medications selected to identify potential allergies were: antihistamines and corticosteroids for systemic use, dermatological preparations with corticosteroids alone or combinations with antiseptics or antibiotics, and finally nasal preparations with corticosteroids or other antiallergic agents. We also investigated the use of antibiotics, emergency room visits, and hospitalizations regardless of the reason for admission.

Statistical analyses were performed using SAS9.4. The influence of gender and age was analyzed using Student’s *t*-test with the significance level set at *p* < 0.01.

## 3. Results

### 3.1. Population and Use of Asthma Medications

For 2018, a total of 441,696 children between the ages of 2 and 18 affiliated to the Independent Health Insurance Funds were included in the study. For 2013 the number was 432,911. Children that were not affiliated continuously to the health insurance fund were excluded, except for deceased children.

As mentioned, we used two different approaches to identify children with asthma: 12.7% of children had at least one dispense of an anti-asthmatic drug in 2013 and 4.6% had at least two dispenses of anti-asthmatic drugs with a minimum of 30 days between two purchases. The results remained relatively stable between 2013 and 2018 (Table 1).

Salbutamol, the only SABA available in Belgium, was the most widely dispensed asthma medication for children in 2018, with 6% of all children in the database receiving it. Ipratropium (SAMA) and budesonide (ICS) were in second and third place, both with 4%. Focusing only on children who received at least one asthma medication, these percentages rose sharply to 51% for salbutamol, 35% for ipratropium, and 32% for budesonide (Table 2).

### 3.2. Influence of Age

**Figure 1:**
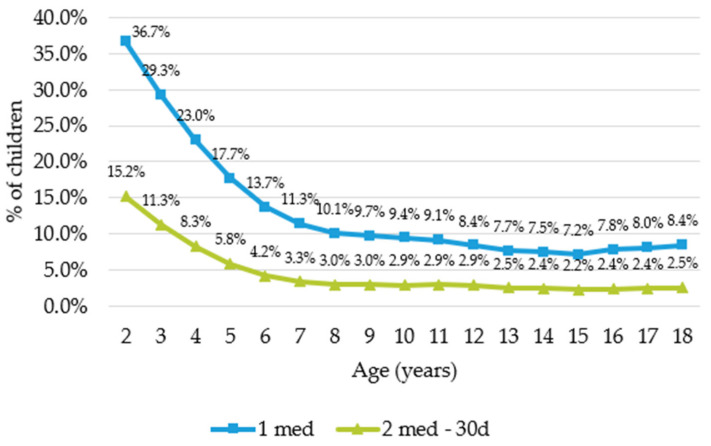
Use of anti-asthmatic drugs in 2018 by age and according to the approach.

Our results showed that preschool children were more likely than older children to have taken asthma medication. (Figure 1) This was statistically significant (*t*-test with *p* < 0.01) when comparing the group of preschool children (2–6 years old) with the group of older children (7–18 years old) for both approaches.

**Figure 2:**
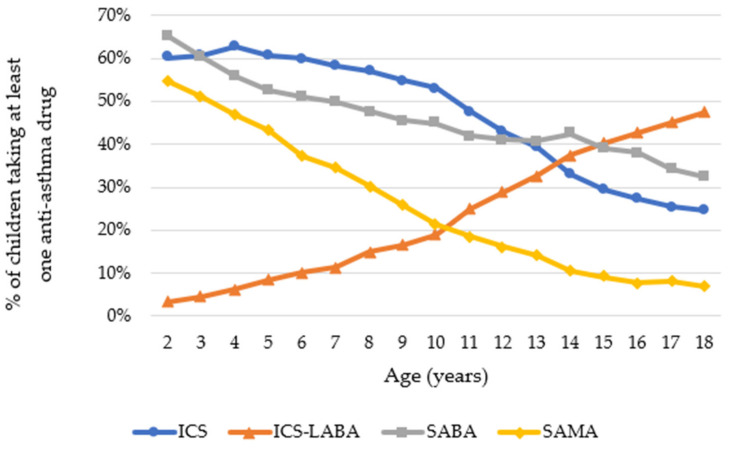
Proportion of children taking asthma medications in 2018 according to pharmacological class.

The use of drug combination ICS-LABA increased with age. In contrast, the use of SABA and SAMA seemed to decrease with age (Figure 2).

Regarding pharmacological classes, ICS, in combination or not with LABA, were the most dispensed drugs among children (65.41%). These were followed by SABA (50.4%) and SAMA (33.6%).

**Figure 3:**
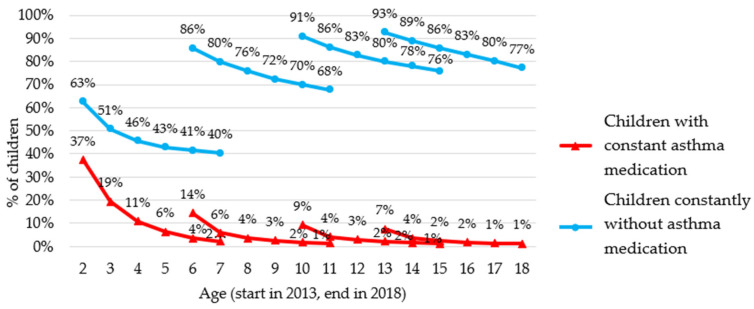
Follow-up between 2013 and 2018 of children aged 2, 6, 10, and 13 years who were not taking asthma medication or were taking at least one in 2013.

Our results indicated that only a limited proportion of children who took asthma medications in 2013 would continue to use them continuously in the following years. For example, in Figure 3, among children who were 2 years old in 2013, 37% received at least one asthma medication that year, but only 2% received asthma medication every year for 5 years. Therefore, only 2% of all 2 year olds in 2013 would have consistently taken asthma medications between 2013 and 2018.

Among younger children, we observed a high initial share of medication users (14, 37%), but a large drop over the years whilst among older children, the initial share of medication users was quite low (7–9%), but a higher share of them would continue to take asthma medication consistently (Figure 3).

**Figure 4:**
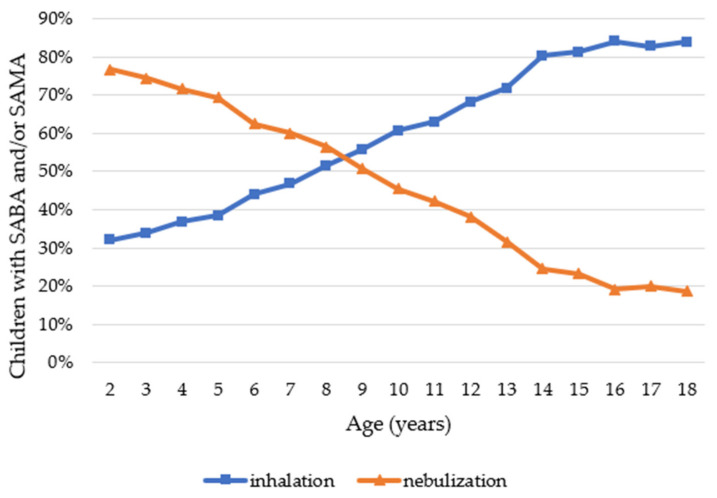
Proportion of children using a nebulizer and/or inhaler among all children who have taken at least one SABA or SAMA.

Regarding the type of administration, nebulization was used in young children more often than inhalation. This trend seemed to reverse around the age of 8 years. Children could take both inhalation and nebulisation medications, which explains why the sum of inhalation and nebulisation is larger than 100% (Figure 4).

### 3.3. Influence of Gender

**Figure 5:**
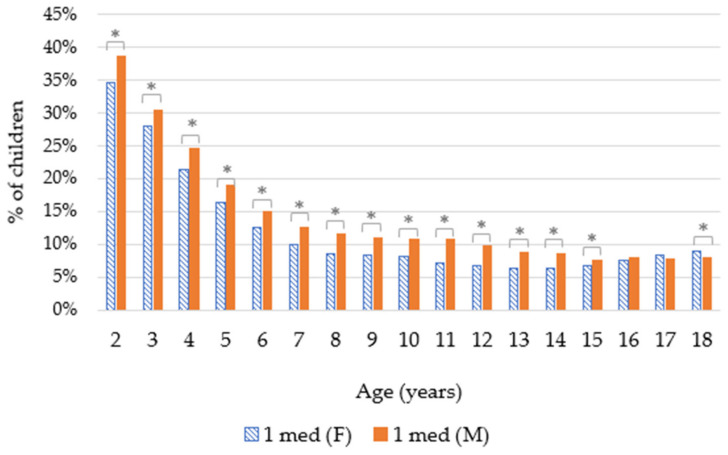
Proportion of children taking asthma medications according to gender (F = female; M = male; 1 med = children with at least one dispense of an anti-asthmatic drug; * = the difference between male and female was statistically significant (*t*-test with *p* < 0.01)).

**Figure 6:**
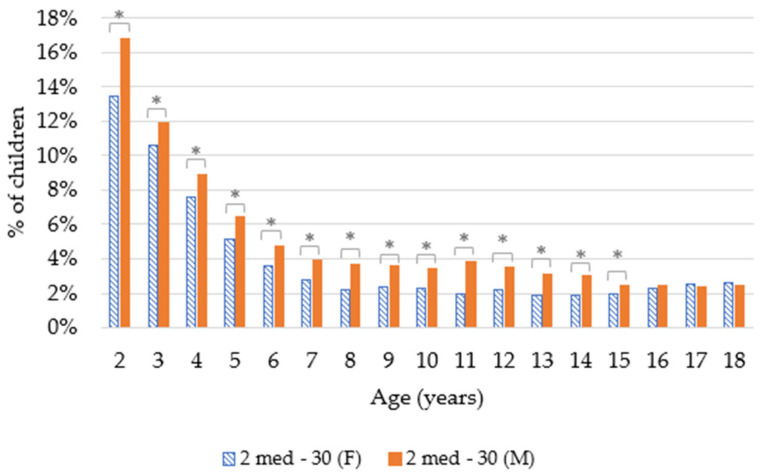
Proportion of children taking asthma medications according to gender (F = female; M = male; 2 med-30 = children with at least two asthma medications with at least 30 days between two purchases; * = the difference between male and female was statistically significant (*t*-test with *p* < 0.01)).

Our results showed that up to the age of 15, a higher percentage of boys than girls were prescribed anti-asthma medications. These differences are statistically significant for both approaches (*t*-test with *p* < 0.01). For children aged 16 and 17, there is no statistically significant difference between girls and boys. Regarding 18 year olds, a slightly higher percentage of girls than boys used these medications, and this is statistically significant for those taking one asthma medication (*t*-test with *p* < 0.01) (Figure 5 and Figure 6).

### 3.4. Factors Associated with Anti-Asthmatic Drugs Utilization

#### 3.4.1. Antibiotics and Allergy Medications

Children who received asthma medication were twice as likely to take allergy medication or antibiotics in 2018 (Table 3).

#### 3.4.2. Emergency Department Visits for Children Based on Asthma Medication Use and Age

Children who received asthma medications were more likely to end up in the emergency room than those who did not in 2018; 30.3% of children with at least one asthma medication and 34.1% of children with at least two dispenses of anti-asthmatic drugs were compared to 19.5% children without asthma medication. As a result, the number of emergency-room visits was twice as high for children taking asthma medication per 1000 children (Table 4).

Children with at least one asthma medication were twice as likely to be hospitalized (with overnight stay) as those without asthma medications. As a result, the average number of hospitalizations was significantly higher for children with asthma medications (Table 5).

## 4. Discussion

### 4.1. Population

In 2018, we observed that 12.9% of children received at least one asthma medication in the year. This percentage was lower (4.4%) for children with at least two dispenses of anti-asthmatic drugs with at least 30 days between two purchases. Our results seemed to remain stable from 2013 to 2018. However, it is difficult to find specific criteria for identifying asthmatic children in our database. With the first group consisting of children who received at least one asthma medication in the year, we may have included children who do not have asthma but received a drug recommended to treat another condition such as symptoms associated with acute respiratory infections [13]. As the second group contained children with at least two dispenses of anti-asthmatic drugs with at least 30 days between two purchases, this allowed us to introduce a certain recurrence in the prescription of anti-asthmatic drugs and thus avoid anti-asthmatics drugs prescribed to treat other pathologies. Indeed, Schmiedl et al. concluded that “off-label prescribing of respiratory drugs is common especially in young children” [13].

According to Achakulwisut et al., the prevalence of asthma in Belgian children between 1 and 18 years was 7.5–10% [12]. It was higher than for Belgian adults (5.8%) [14]. A Danish study that attempted to optimize a methodology for the study of asthmatic children used the same criteria as our study [15]. Among children aged 6–14, they selected those who had received at least one prescription for asthma medication. They found that 7.7% of children between 6–14 years received at least one asthma medication. An Italian study observed that 5.1% of 6–17 years olds received at least one asthma medication, while our results were 8.7% of 7–18 years olds [16].

As shown in Table 2, salbutamol (SABA) was the most widely dispensed asthma medication for children. This meant that SABA was sometimes used in monotherapy as a reliever according to GINA (Global Initiative for Asthma) guidelines in progress at the time of the study [17]. Since 2019, GINA guidelines no longer recommend SABA as the preferred reliever from 12 years old. Currently, the preferred initial treatment is a low-dose ICS-formoterol to use when needed, and the use of SABA should be used as the alternative. Indeed, the use of ICS-formoterol as a reliever would reduce the risk of severe exacerbations compared to SABA. For children between 6 and 11 years old, SABA can be used as a reliever but not as monotherapy, since low doses of ICS are recommended whenever SABA are taken [1]. The high use of SAMA, only rarely mentioned in guidelines, can be questioned as well. It can be used, in a minority of cases, for an additive bronchodilator effect to SABA or as an alternative in the case of contraindication to SABA [1]. The only monoclonal antibody available on the market in Belgium for the duration of the study was omalizumab. This molecule seems to be rarely prescribed in Belgium. Indeed, in 2018, it was prescribed to less than 0.03% of children with at least one asthma medication.

### 4.2. Influencing Factors

#### 4.2.1. Age

For both methodologies we observed a decrease in prevalence with age. The consumption of asthma medications in young children (2–6 years old) was almost three times higher than in older children (7–18 years old).

The use of asthma medications in other conditions such as bronchiolitis, wheezing, or cough could explain these higher results in younger children [18]. These diseases are quite common among children. For example, about a third of children aged 3 years have already had one episode of wheezing, and this percentage is close to 50% by the age of 6 years [19,20].

The decrease in the use of asthma medications in the older age group could be explained by the possibility of confirming the diagnosis of asthma through functional tests around the age of 6 [19].

#### 4.2.2. Gender

As shown in Figure 5 and Figure 6, up to the age of 15 years, asthma medications were prescribed more often to boys than to girls. Different studies have been shown to have the same trend; for example, a meta-analysis showed that boys were more often associated with asthma (OR 1.7) [21]. This could be explained by the diameter of the airways of young boys in relation with their lung volume, which is smaller than for girls. Due to this, young boys are more likely to present airway obstruction [22]. This trend seemed to reverse after 15 years old and seemed to continue among adults. This could be explained by hormonal factors, as explained by Zein et al., “estrogen signaling in airway inflammation can contribute to gender differences in asthma” [23]. In Belgium, according to Van der Heyden and Charafeddine, the prevalence of asthma was higher in women (6.5%) than in men (5%) for those over 15 years of age [14].

#### 4.2.3. Type of Administration

There are different ways to administer inhaled medications, such as bronchodilators: by nebulization or by inhalation. Two different types of devices exist for inhalation: pressurized metered dose inhalers (pMDIs) or dry powder inhalers (DPIs).

In Belgium, short-acting bronchodilators, including the most prescribed molecule (salbutamol), administered by DPI are not reimbursed, so they did not appear in our database. Therefore, we have compared pMDI inhalation administration with nebulization.

Our results showed that up to the age of 8 years, children received medication more often by nebulization rather than inhalation (Figure 4), despite guidelines that recommend using inhalation as much as possible [1].

Indeed, during administration by nebulization, the device will transform liquids into fine particles that will be inhaled during tidal breathing. This type of administration is generally used in young children since it is not dependent on a patients’ ability to coordinate inhalation with actuation. However, a major disadvantage of this method is the low lung deposition due to loss during breathing and the size of the particles emitted. Another concern about pMDIs is their impact on the environment. Indeed, these devices use hydrofluorocarbons known contribute to greenhouse gases [24].

For inhalation devices, especially pMDIs, the size of the particles emitted is finer than for nebulization. On the other hand, due to the speed at which the drug is released from the device, there is a significant deposition of aerosol particles in the oro-pharyngeal region. This phenomenon can be reduced using expansion chambers, which also allows the use of pMDI in patients who do not have good hand-to-mouth coordination, such as children. Regarding DPIs, they do not require good hand-to-mouth coordination, but the dose released, and the size of the particles emitted will depend on the patient’s inspiratory flow [25,26].

It would be interesting to analyze the consumption of asthma medications according to other factors. For example, the body mass index, ethnic origin, or medication adherence could be linked with the consumption of asthma medications [27,28]. Unfortunately, we do not have access to this information in our database.

### 4.3. Factors Associated with Anti-Asthmatic Drugs Utilization

#### 4.3.1. Antibiotics and Allergy Medications

A Belgian study that analysed medication dispensing data from 2005 to 2007 showed that children who received asthma medications are 1.9 times more likely to receive antibiotics than those without asthma medications [29].

Our results showed that this trend was more pronounced in the youngest age group (2–6 years old); 67.5% to 75.6% of those taking asthma medications received antibiotics vs. 34.8% for children without asthma medications. These results decreased from 50.1% to 51.3% vs. 25.6% for older children (7–18 years).

These results could be explained by the frequent occurrence of wheezing in young children. Indeed, one child out of two has already had a wheezing episode before the age of 6 [30]. Antibiotics are often prescribed to treat it, although in most cases it is a viral infection. Several studies have already shown that the use of antibiotics in this kind of pathology is not recommended and has not shown a positive effect [31,32].

According to Baan et al., the most frequent reasons for use of antibiotics in patients with asthma were bronchitis and asthma exacerbations when antibiotics are not recommended [33]. The use of antibiotics for the treatment of upper and lower respiratory tract infections was more common with asthmatic children than for non-asthmatic children. However, most of these infections are caused by viruses, so antibiotics are not required.

As with antibiotics, our results showed that children who took asthma medications were more likely to additionally take allergy medications. For example, 17.6% of children without asthma medications received allergy medications vs. 36.1% for children with at least one asthma medication. The same trend was observed for the older group and the results were even higher for children with two prescriptions of asthma medications (and 30 days between dispenses). Some of the underlying causes of asthma cause other allergic manifestations, such as food allergies, rhino-conjunctivitis, or eczema. Different allergies can lead to the appearance of asthma symptoms (pollens, dust mites, molds, animals, etc.). These mechanisms play a role in about half of asthmatic children [4]. Allergic rhinitis, known as “hay fever”, is very common in children with asthma and with proper treatment, asthma can be better controlled [34].

#### 4.3.2. Emergency Room Visits and Hospitalizations

Children who received asthma medications were more likely to end up in the emergency room than those who did not: 30.3% vs. 19.5% in 2018. The average number of emergency room visits in 2018 was significantly higher; 1.3 on average for children who did not take asthma medication and 1.5 for those who did. These differences led to a total of twice as many visits per 1000 children for children taking asthma medication in 2018: 468 vs. 258 per 1000 children. Our database did not allow us to deduce the specific reason for the emergency visit. One reason for hospitalization could be severe asthma attacks. In young children, these attacks are often caused by (viral) infections of the airways, such as a common cold. Inhaled allergens (dust, dust mites, pollen, mold), inhaled air pollutants such as tobacco smoke and exhaust fumes, medications, exercise, emotional stress, and even certain foods and drinks could cause an asthma attack [4]. Attacks are characterized by one or more of the following symptoms: increasing shortness of breath, coughing, wheezing, and chest tightness. Some of these attacks can be controlled at home, others cannot. Several studies showed that as a result, children who were taking asthma medications were more likely to end up in the emergency room and hospital than those who were not [35,36,37]. A Danish study showed that children with asthma were three times more likely to end up in hospital than those without [15].

In our study, children who were prescribed at least one medication were twice as likely to be hospitalized (overnight) as those who were not prescribed asthma medication. We also found that the average number of hospitalizations was higher. Among children who received asthma medications, these medications were used in more than 3 out of 10 hospitalizations, indicating that asthma (or its symptoms) played a role.

Studies showed that the percentage of hospitalizations due to asthma in Belgium (2.91%) was higher than the average in Europe (2.09%). This could indicate that primary care and continuity of care could be improved [4,38].

### 4.4. Strengths and Weaknesses

Caution is required in interpreting the results. Indeed, the identification of asthma patients was uncertain since we did not have access to the diagnosis. We could only rely on drug dispensing, but the drugs could be used to treat other diseases than asthma. Therefore, we could not be sure that the patients we had identified as “asthmatics” really were asthmatic.

In addition, we only had access to dispense data. This means that the drug must have been prescribed and the patient must have picked it up at the pharmacy, otherwise the data did not appear in the database. The limitation of our method is that we may not have data on the most disadvantaged populations who for economic reasons would not have purchased the drugs.

However, our database from the Independent Health Insurance Funds represents about 20% of the Belgian population, which is not negligible. Thanks to the data, we were able to investigate the risk factors related to the consumption of asthma medications and can suggest several recommendations.

### 4.5. Recommendations

According to our results, nebulization was the main mode of administration for SABA and SAMA in young children, despite GINA recommendations, which advocate inhalation with a spacer device. Nebulization is reserved for a minority of children who “cannot be taught effective use of a spacer device” [1]. In addition, children and parents need to be well informed about the proper use of inhalation devices. Between 70 and 80% of patients do not have proper inhaler technique. This education is the responsibility of all health care providers involved with the child: general practitioner, pediatrician, pharmacist, etc. and should be repeated regularly. The task of health care providers can be facilitated by providing educational tools adapted to children and their parents, such as written material or videos [39].

Furthermore, a written action plan should be developed for each child with asthma. This is a set of personalized instructions for the child and family regarding the monitoring and management of the disease. This plan can help the child and family members to better manage the disease and avoid emergency-room visits or hospitalizations. The plan should include at least the following:

- the patient’s usual asthma medications;
- when and how to increase medication use and give oral corticosteroids;
- how to obtain access to medical care if treatment is not working.

It would be interesting to advance research into the use of other monoclonal antibodies in children, as most are only available for adults. Indeed, a good control of asthma in children avoids the appearance of complications and improve the prognosis of these patients in adulthood [8].

In addition to drug treatments, there are several other interventions that have a beneficial effect on asthma. For example, reducing exposure to identified allergens and air pollution. It is important that the mother does not smoke during pregnancy. Moreover, the child should not be exposed to smoke, as this increases the risk of hospitalizations and poor asthma control [1].

## 5. Conclusions

Asthma is a common disease in children, which is suggested by the results of our study. Inhaled corticosteroids were the therapeutic class most used by children. SABA was sometimes used in monotherapy, according to current GINA guidelines at the time of the study. The high use of SAMA, only rarely mentioned in guidelines, could be questioned as well. Our results indicated that young children mainly used a nebulizer despite the recommendation to use inhalation with a spacer.

This study told us that children who took asthma medications had more health problems than other children: they were more likely to receive allergy medications or antibiotics, and they were more likely to visit the emergency room and be hospitalized. All these elements have a significant financial impact. Moreover, this chronic disease has a social and psychological impact on the child and his family.

Further studies are needed to understand the reasons for this increased use of health care, which could be related, for example, to medication adherence problems.

## Figures and Tables

**Table 1:** Overview of the use of asthma drugs in 2013 and 2018 for children affiliated to the Independent Health Insurance Funds.

	2013			2018		
Age	Number of Children (n)	≥1 Asthma Medication	≥2 Asthma Medications and 30 Days	Number of Children (n)	≥1 Asthma Medication	≥2 Asthma Medications and 30 Days
2–6 years	124,443	23.7%	9.1%	121,739	23.8%	8.8%
7–18 years	308,468	8.2%	2.8%	319,957	8.7%	2.7%
Total	432,911	12.7%	4.6%	441,696	12.9%	4.4%

**Table 2:** Top five most dispensed asthma medications in 2018.

	Molecule	Pharmacological Class	ATC Code	Number of Children	% of All Children	% of All Children with at Least One Asthma Medication
1	Salbutamol (inhalation)	SABA	R03AC02	28,372	6%	51%
2	Ipratropium	SAMA	R03BB01	19,301	4%	35%
3	Budesonide	ICS	R03BA02	17,522	4%	32%
4	Fluticasone	ICS	R03BA05	12,216	3%	22%
5	Montelukast	Leukotriene Receptor Antagonists	R03DC03	8013	2%	14%

**Table 3:** Use of antibiotics and allergy medications by children in 2018, according to asthma medications.

Age	Studied Group	Antibiotics	Allergy Medications
2–6 years	No asthma medication	34.8%	17.6%
	≥1 asthma medication	67.5%	36.1%
	≥2 asthma medications in 30 days	75.6%	45.2%
7–18 years	No asthma medication	25.6%	16.8%
	≥1 asthma medication	50.1%	49.0%
	≥2 asthma medications in 30 days	51.3%	66.1%

**Table 4:** Emergency-department visits for children based on asthma medication use and age.

Age	Studied Group	Children with at Least One ER Visit	Number of ER Visits (for 1000 Children)
2–6 years	No asthma medication	23.0%	318
	≥1 asthma medication	34.8%	561
	≥2 asthma medications in 30 days	38.8%	668
7–18 years	No asthma medication	18.4%	239
	≥1 asthma medication	25.6%	371
	≥2 asthma medications in 30 days	28.3%	559
Total	No asthma medication	19.5%	258
	≥1 asthma medication	30.3%	468
	≥2 asthma medications in 30 days	34.1%	559

**Table 5:** Hospitalizations in children according to asthma medications and age.

Age	Studied Group	% of Hospitalizations	Average Numbers of Hospitalizations (with Overnight Stay)
2–6 years	No asthma medication	4%	1.21
	≥1 asthma medication	12%	1.33
	≥2 asthma medications in 30 days	16%	1.40
7–18 years	No asthma medication	3%	1.29
	≥1 asthma medication	6.3%	1.39
	≥2 asthma medications in 30 days	8.0%	1.50
Total	No asthma medication	3.4%	1.27
	≥1 asthma medication	8.9%	1.35
	≥2 asthma medications in 30 days	12.5%	1.43

## Data Availability

The data presented in this study are available on request from the corresponding author.
